# Establishment of a prognosis Prediction Model Based on Pyroptosis-Related Signatures Associated With the Immune Microenvironment and Molecular Heterogeneity in Clear Cell Renal Cell Carcinoma

**DOI:** 10.3389/fonc.2021.755212

**Published:** 2021-11-05

**Authors:** Aimin Jiang, Jialin Meng, Yewei Bao, Anbang Wang, Wenliang Gong, Xinxin Gan, Jie Wang, Yi Bao, Zhenjie Wu, Juan Lu, Bing Liu, Linhui Wang

**Affiliations:** ^1^ Department of Urology, Changhai Hospital, Naval Medical University (Second Military Medical University), Shanghai, China; ^2^ Department of Urology, The First Affiliated Hospital of Anhui Medical University, Institute of Urology, Anhui Medical University, Anhui Province Key Laboratory of Genitourinary Diseases, Anhui Medical University, Hefei, China; ^3^ Department of Urology, Changzheng Hospital, Naval Medical University (Second Military Medical University), Shanghai, China; ^4^ Vocational Education Center, Naval Medical University (Second Military Medical University), Shanghai, China; ^5^ Department of Urology, The Third Affiliated Hospital, Naval Medical University (Second Military Medical University), Shanghai, China

**Keywords:** clear cell renal cell carcinoma, pyroptosis, immune microenvironment, single cell, prognosis, drug response

## Abstract

**Background:**

Pyroptosis is essential for tumorigenesis and progression of neoplasm. However, the heterogeneity of pyroptosis and its relationship with the tumor microenvironment (TME) in clear cell renal cell carcinoma (ccRCC) remain unclear. The purpose of the present study was to identify pyroptosis-related subtypes and construct a prognosis prediction model based on pyroptosis signatures.

**Methods:**

First, heterogenous pyroptosis subgroups were explored based on 33 pyroptosis-related genes and ccRCC samples from TCGA, and the model established by LASSO regression was verified by the ICGC database. Then, the clinical significance, functional status, immune infiltration, cell–cell communication, genomic alteration, and drug sensitivity of different subgroups were further analyzed. Finally, the LASSO-Cox algorithm was applied to narrow down the candidate genes to develop a robust and concise prognostic model.

**Results:**

Two heterogenous pyroptosis subgroups were identified: pyroptosis-low immunity-low C1 subtype and pyroptosis-high immunity-high C2 subtype. Compared with C1, C2 was associated with a higher clinical stage or grade and a worse prognosis. More immune cell infiltration was observed in C2 than that in C1, while the response rate in the C2 subgroup was lower than that in the C1 subgroup. Pyroptosis-related genes were mainly expressed in myeloid cells, and T cells and epithelial cells might influence other cell clusters *via* the pyroptosis-related pathway. In addition, C1 was characterized by MTOR and ATM mutation, while the characteristics of C2 were alterations in SPEN and ROS1 mutation. Finally, a robust and promising pyroptosis-related prediction model for ccRCC was constructed and validated.

**Conclusion:**

Two heterogeneous pyroptosis subtypes were identified and compared in multiple omics levels, and five pyroptosis-related signatures were applied to establish a prognosis prediction model. Our findings may help better understand the role of pyroptosis in ccRCC progression and provide a new perspective in the management of ccRCC patients.

## Introduction

Renal cell carcinoma (RCC) is a common urologic malignancy with an incidence only secondary to prostate and bladder cancer (1). According to the characteristics of molecular biology and histopathology, RCC can be categorized into two main types: clear cell renal cell carcinoma (ccRCC) and non-clear cell renal carcinoma (nccRCC) including papillary renal cell carcinoma (pRCC), chromophobe cell renal cell carcinoma (cRCC), and collecting duct renal cell carcinoma (cdRCC) (2). Among them, ccRCC accounts for approximately 75–80%. As only 6–10% of the patients developed typical symptoms like backache, an abdominal mass, or hematuria, it is difficult to diagnose RCC in the early stage (3). As ccRCC is insensitive to conventional chemotherapy and radiotherapy, nephrectomy, target therapy, and immunotherapy are the mainstays of treatment for ccRCC (4). But as ccRCC is an extremely heterogeneous disease, even patients with similar clinical characteristics who received similar treatments may have distinctive outcomes (5). Hence, it is urgent to explore the innate mechanism of ccRCC for the sake of developing novel therapeutic strategies for improving the overall clinical outcome of this disease.

Cell death, which includes apoptosis, necrosis, autophagy, anoikis, and pyroptosis, is a particular mechanism regulating stress response, cell proliferation, homeostasis, and tumor progression (6). Pyroptosis as an important anticancer defense mechanism has been deeply and extensively studied, while the relationship between ccRCC and pyroptosis remains unclear. Pyroptosis is an inflammatory form of cell death triggered by certain inflammasomes, leading to the cleavage of gasdermin D (GSDMD) and the activation of inactive cytokines interleukin-18 (IL-18) and IL-1β. Recently, extensive studies have focused on elucidating the molecular mechanism underlying pyroptosis as well as the mechanism of inducing pyroptosis in tumor cells (7). Tan et al. reported that the knockdown or inhibition of BRD4, a member of BET family, could activate the NF-κB-NLRP3-caspase-1 pyroptosis signaling pathway, thus attenuating the cell proliferation and epithelial-mesenchymal transition (EMT) of clear cancer cell lines (8). All these findings suggest that pyroptosis play an essential role in the progression and therapy in ccRCC, and comprehensive analysis of pyroptosis may shed new light on the development of strategies for the treatment of ccRCC. However, the accurate mechanism of pyroptosis in ccRCC has been less studied. Herein, we aimed to perform a systematic research to compare the expression level in ccRCC and normal renal tissue, decipher the role of pyroptosis in the ccRCC microenvironment, and construct a pyroptosis-related risk model for ccRCC.

## Materials and Methods

### Public Dataset Collection

ccRCC data were enrolled in public databases [including The Cancer Genome Atlas (TCGA) and the International Cancer Genome Consortium (ICGC)]. For datasets in public databases, institutional review board approval and informed consent were not required. Level-3 transcriptome and clinical information were downloaded from the TCGA and ICGC. RNA-seq data of count and FPKM normalized from the TCGA–KIRC cohort were obtained from the GDC database (https://portal.gdc.cancer.gov/); the former was utilized for different expression analysis and later was further transformed to log2 (TPM+1) for further analysis. All expression data have been normalized before analysis. Patients were excluded if they 1) did not have prognostic information and 2) died within 30 days. The overall workflow of this research is displayed in **Figure S1**.

### Identification of Differentially Pyroptosis Status in ccRCC

Altogether, 33 pyroptosis-related genes were retrieved from prior articles and reviews (**Table S1**). Correlations between these pyroptosis-related genes were assessed by Spearman’s rank correlation using the R “corrplot” package. The cluster analysis of pyroptosis-related genes was performed by hclust and kmeans algorithms. Then, 531 ccRCC patients were categorized into different subgroups using PCA, and finally the subtype number k = 2 was selected in that it turned out to be the best classifier number. The R package “DEseq2” was employed to identify DEGs between different groups, with the threshold set as *p*-adjusted value < 0.01 and abstract log-fold change = 2. To explore the potential molecular mechanisms underlying the subgroups, the R package “clusterProfiler” was used to perform gene ontology (GO), Kyoto Encyclopedia of Genes and Genomes (KEGG) pathway, and Gene set enrichment analysis (GSEA).

### Analysis of the DEGs

The R “DEseq2” package was applied to identify DEGs between different groups, with the threshold set as *p*-adjusted value < 0.01 and abstract log-fold change = 2. The R “clusterProfiler” package was used to explore the potential molecular mechanisms of DEGs, including gene ontology (GO), Kyoto Encyclopedia of Genes and Genomes (KEGG) pathway, and Gene set enrichment analysis (GSEA). The Cytoscape plugin “iREgulon” was employed to analyze the transcription of the down- and upregulated genes. Difference in signaling pathways between C1 and C2 subgroups was presented by adoption of the gene set from the MSigDB database (9). The potential transcription factors for DEGs were analyzed by using the module “iRegulon” from the Cytoscape software.

### Differences in Tumor Microenvironment (TME) and Immunotherapy Response

To quantify the proportion of immune cells between the subtypes, several immune-related algorithms including TIMER, CIBERSORT, QUANTISEQ, MCPCOUNTER, XCELL, and EPIC were employed to calculate the cellular components or immune cell enrichment scores in ccRCC tissues, and differences between C1 and C2 subgroups were compared. Single sample gene set enrichment analysis (ssGSEA) was employed to quantify the infiltration abundance of immune cells in ccRCC TME (10–13). Differences in immune cell infiltration in TME were visualized by Heatmap and boxplot. The R ESIMATE package was used to identify the stromal component and the immune component between the two subgroups. Tumor Immune Dysfunction and Exclusion (TIDE) algorithms were applied to predict the response rate of the immune checkpoint inhibitor response in ccRCC.

### Cell-Cell Interaction Analysis

PRJNA705464, which is a large database containing cells totally, was applied to investigate the role of pyroptosis-related genes in cell–cell interaction in ccRCC TME. Only untreated tumor samples from PRJNA705464 were applied for further analysis. phs002252.v1.p1, which is another single cell dataset including 13 ccRCC patient tissue samples, was used to compare the different expression levels of pyroptosis-related genes among different clinical stages. The R “Seurat” package was used for dimension reduction and clustering analysis, and the R “SingleR” package was introduced for cell type identification (14). The R package “CellChat” and the software “cellphonedb” were applied for cell–cell interaction analysis, and cell–cell interactions based on the expression of reported ligand–receptor pairs in different cell were calculated (15, 16).

### Multi-Omics Data Analysis

Mutation and copy number variations of ccRCC were downloaded from the TCGA database. WES data were used to compare differences in somatic mutation between C1 and C2 using the R “maftools” and “MOVICS” package (17, 18). The co-occurrence and mutually exclusive mutations were identified using the CoMEt algorithms. For copy number variation data, the “GISTIC 2” software in GenePatterns was used to identify significantly deleted or amplified broad and focal segments (19).

### Construction and Validation of the Pyroptosis-Related Risk Scores in Public Data Sets

Univariate Cox regression analysis was firstly applied to assess the prognostic value of the pyroptosis-related genes, and 17 genes were selected out for further analysis. Next, lasso-Cox algorithm was applied to narrow down the candidate genes to develop a robust and concise prognostic model. Ultimately, a five-gene risk model was constructed, and the penalty parameter was decided by 1se with the R “glmnet” package. After standardization and normalization of the TCGA ccRCC expression data, the risk score of each patient was calculated using the following equation: Risk score = 0.0271113*AIM2+0.04147645*GSDMB+0.01748664*IL6+0.01968024*PYCARD-0.08678271*TIRAP (Risk score=Σ*_i_^5^X_i_Y_i_*). Then, each patient from the TCGA and ICGC databases was assigned to a high- or low-risk group based on the median value of the risk score. Kaplan–Meier survival curves were depicted to predict the clinical outcomes in the two groups by the R “survival” package. The ROC curves were depicted, and the area under the curves (AUC) for 0.5-, 1-, 2-, 3-, and 5-year overall survival (OS) and progression-free interval (PFI) were calculated using the R “timeROC” package.

### Assessment of Clinical Significance of the Pyroptosis Subtypes

Clinical characteristics including age, gender, grade, AJCC stage, TNM, OS, and PFI were compared between C1 and C2 subtypes by the R “compare” package. Sensitivity to several chemotherapy drugs was compared by the R “pRRohetic” package (20). IC50 (half maximal inhibitory concentration) values of C1 and C2 subgroups were estimated by ridge regression. The sensitivity of the two subgroups to immune checkpoint inhibitor therapy was predicted by the TIDE (http://tide.dfci.harvard.edu) algorithm. The Genomics of Drug Sensitivity in Cancer (GDSC) database (https://www.cancerrxgene.org) was applied to screen the potential drug for the high-risk subgroup by the R “pRRphetic” package (21).

### Validation of Risk Model-Related Gene Expression in the SMMU Cohort

According to the expression of prognostic genes in the gene signature in the TCGA and ICGC databases, we selected five hub genes (PYCARD, AIM2, IL6, GSDMB, and TIRAP) that were differentially expressed between the cancer and normal tissues for validation using quantitative real-time PCR (RT-qPCR). Informed consent about the tissue sample analysis was obtained from each patient before the initiation of the study, and the study protocol was approved by the Institutional Review Board of the Second Military Medical University (SMMU) Cancer Center. A total of 40 paired normal and cancer tissues were used to validate the different expression level of model-related genes. For detailed experiment procedures, please refer to the previous literature published by our laboratory. The primer sequences used are listed in **Table S2** (see **Supplementary Data**). Immunohistochemical (IHC) staining was performed on Changzheng ccRCC tissue microarray using antibody purchased from the Abcam company (PYCARD, ab283684; AIM22, ab93015; IL6, ab9324; GSDMB, ab235540; TIRAP, ab17218; diluted at 1:100–1:200). The Oncomine database (https://www.oncomine.org/resource/login.html) was utilized to validate the different expression of model-related genes in ccRCC and normal tissue.

### Statistical Analysis

Differences in the expression of the pyroptosis-related genes in the public data sets were compared by one-way ANOVA, and differences in clinical information and immune checkpoint inhibitor response between the two different subgroups were compared by chi-squared test. Differences in OS and PFI between the two subgroups were compared by Kaplan–Meier and log-rank test. Hazard ratios (HRs) were calculated by univariate and multiple Cox regression analysis. The receiver operating characteristic (ROC) curves were plotted by the R “timeROC” package. The performance of the risk score in predicting OS and PFI was evaluated by the area under the ROC curve (AUC) and Harrell’s concordance index (C-index). All P-values were two-sided, with P < 0.05 statistically significant. Benjamini–Hochberg (BH) multiple test correction was used to calculate the adjusted P value. All data processing, statistical analysis, and plot were conducted within the R software (version 4.1.0).

## Results

### Landscape of Pyroptosis Genes in ccRCC

**Figure 1:**
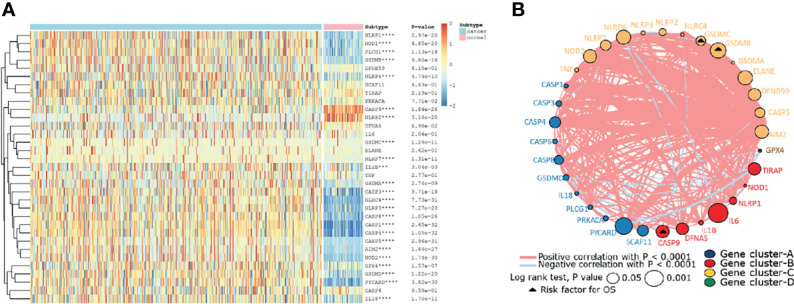
The landscape of pyroptosis-related genes in ccRCC. **(A)** The expression levels of 33 pyroptosis-related genes in ccRCC. The darker color indicates a higher expression, where upregulated genes were marked as red, and downregulated genes were marked as blue. **(B)** Interaction of pyroptosis-related genes. Gene cluster A, blue; gene cluster B, red; gene cluster C, yellow; gene cluster D, green. The circle size represents the effect of each gene on prognosis, and the range of values was calculated by log-rank test as p<0.05 and p<0.001. The triangle dots represent the risky factors. The lines linking genes indicate their interactions, and thickness represents the correlation strength between genes. Blue lines represent negative correlation, while red lines represent positive correlation.

Firstly, the expression levels of 33 pyroptosis genes were compared between the ccRCC and normal renal tissues. The result showed that the expression level of NLRP1, NOD1, PLCG1, PLCG1, GSDMB, NLRP6, GSDMC, NLRP7, IL1B, GSDMA, CASP3, NLRRC4MNLRP3, CASP8, CASP1, CASP4, CASP5, AIM2, NOD2, GPX4, GSDMD, PYCARD, and IL18 in the ccRCC tissues was higher than that in the normal renal tissues, while the expression level of genes containing CASP9 and NLRP2 in the normal renal tissues was higher than that in the ccRCC tissues (**Figure 1A**). To further explore the interaction and correlation of the pyroptosis genes, we constructed a comprehensive network and divided the genes into four clusters. It was found that three pyroptosis genes were risk prognosis factors (**Figure 1B**).

### Two Clusters of ccRCC Are Identified by Consensus Clustering of Pyroptosis Genes

**Figure 2:**
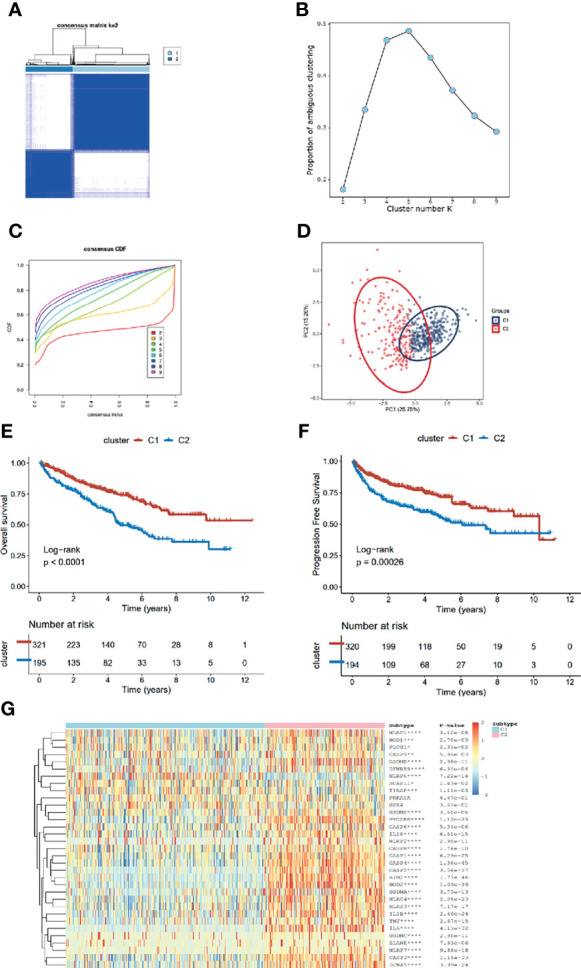
Identification of pyroptosis clusters of ccRCC. **(A)** Consensus cluster matrix of ccRCC tumor samples when k =2. **(B)** The proportion of ambiguous clustering score, and the optimal k number turns to 2. **(C)** The cumulative distribution function (CDF) curves also indicated k=2 as the optimal cluster number. **(D)** Two-dimensional principal component plot by matrix containing 33 pyroptosis-related genes expression from the ccRCC cohort. The blue dots represent C1, and the red dots represent C2. **(E, F)** Kaplan–Meier analysis for overall survival (left) and progression-free interval of the two subtypes in the TCGA cohort. **(G)** The expression heatmap of the 33 pyroptosis-related genes in the two subtypes.

After removing the normal renal tissues, we used unsupervised clustering methods to classify the tumor samples into different molecular subgroups based on pyroptosis-related genes. The optimal cluster number was identified by the R “ConsensusClusterPlus” package, and the clustering stability was evaluated by the proportion of the PAC algorithm. Finally, two distinct clusters, termed as C1 and C2, were identified (**Figures 2A–D**). To evaluate the clinical significance of subtypes, clinical outcomes, and clinicopathological features, differences in survival in terms of OS and PFI were compared between the two clusters by log-rank test and Kaplan–Meier curve (**Figures 2E, F**). In addition, we found that most pyroptosis-related genes were highly expressed in C2 as compared with C1 (**Figure 2G**). Compared with C1, C2 was significantly correlated with a higher grade, AJCC score, and TNM status (**Table 1**).

**Table 1:** Difference of clinical characteristics between C1 and C2 subgroups.

	C1	C2	p. overall
	***N=324***	***N=197***	
Age	60.2 (12.2)	61.2 (12.1)	0.325
Gender:			0.032*
Female	125 (38.6%)	57 (28.9%)	
Male	199 (61.4%)	140 (71.1%)	
Grade:			<0.001*
G1	14 (4.32%)	0 (0.00%)	
G2	164 (50.6%)	60 (30.5%)	
G3	120 (37.0%)	86 (43.7%)	
G4	22 (6.79%)	50 (25.4%)	
GX	4 (1.23%)	1 (0.51%)	
AJCC:			<0.001*
Stage I	193 (59.6%)	69 (35.0%)	
Stage II	35 (10.8%)	21 (10.7%)	
Stage III	61 (18.8%)	62 (31.5%)	
Stage IV	35 (10.8%)	45 (22.8%)	
N:			0.011*
N0	145 (44.8%)	92 (46.7%)	
N1	4 (1.23%)	11 (5.58%)	
NX	175 (54.0%)	94 (47.7%)	
M:			<0.001*
M0	269 (83.0%)	148 (75.1%)	
M1	32 (9.88%)	43 (21.8%)	
MX	23 (7.10%)	6 (3.05%)	
OS	0.24 (0.43)	0.47 (0.50)	<0.001*
PFI	0.24 (0.43)	0.40 (0.49)	<0.001*

*P value <0.05 is considered statistically significant. Summary descriptive table by groups of “Cluster”.

### Identification of DEGs and Functional Analysis

**Figure 3:**
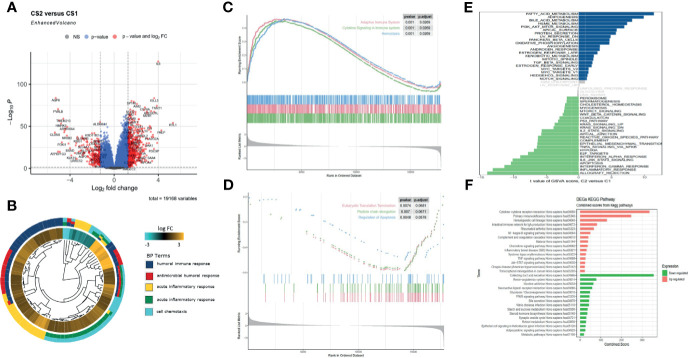
Functional enrichment analysis of DEGs between C1 and C2 subtypes. **(A)** Volcano map of differentially expressed genes. **(B)** GO enrichment analysis. **(C, D)** GSEA analysis shows the hallmarks between the subgroups. **(E)** Gene set enrichment analysis (GSVA) shows the significant enrichment differences between the subgroups. **(F)** KEGG pathway analysis.

The gene expression profiles of ccRCC were analyzed to identify pyroptosis-related DEGs, including the upregulated and downregulated ones in C2 relative to C1 (**Figure 3A**). Then, DEGs were used to perform enrichment analyses. The GO results demonstrated that the DEGs were enriched in humoral immune response, receptor ligand activity, and collagen-containing extracellular matrix (**Figures 3B**, **S2A**). Transcription factor analysis of the downregulated and upregulated genes was conducted using “iRegulon.” The transcriptional regulation network of these down- and upregulated genes is shown in **Figure S2B**. GSEA analysis showed that the adaptive immune system, cytokine signaling in the immune system, and hemostasis were upregulated, while eukaryotic translation termination, peptide chain elongation, and regulation of apoptosis were downregulated in C2 *vs.* C1 (**Figures 3C, D**). The TF of upregulated genes was REST, and for the downregulated genes, it was IKZF2. GSVA analysis indicated that fatty acid metabolism, adipogenesis, and PI3K-Akt-mtor pathway were upregulated in C2, while inflammatory response, apoptosis, and IL6-JAK-STAT3 pathway were upregulated in C1 (**Figure 3E**). The KEGG results demonstrated that cytokine receptor interaction and primary immunodeficiency were upregulated, while collecting duct acid secretion was downregulated in C2 relative to C1 (**Figure 3F**). Since the C1 and C2 subgroups were identified in the TCGA database, to validate the consistency of cluster analysis based on pyroptosis, ccRCC from the ICGC database was utilized to examine such consistency. Interestingly, the subgroup in ICGC highly matched in subgroups in TCGA, and C1 and C2 subgroups showed similar pyroptosis state and prognostic characteristics in the TCGA database (**Figure S2C**).

### Comparison of the Immune Landscape Between the Subgroups

**Figure 4:**
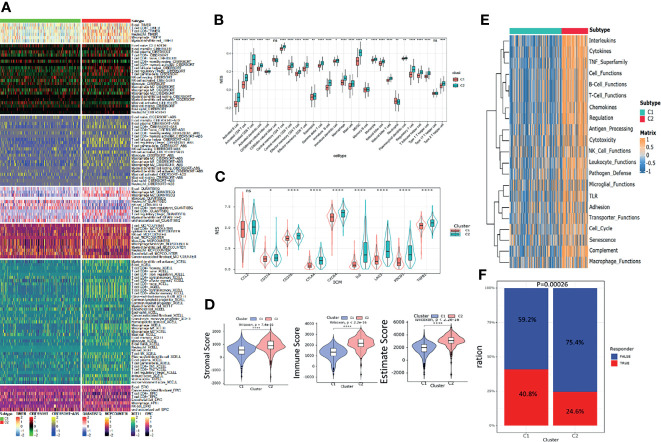
Immune landscapes between the pyroptosis subgroups. **(A)** Heatmap of tumor -related infiltrating immune cells based on TIMER, CIBERSORT, CIBERSORT-ABS, QUANTISEQ, MCPcounter, XCELL, and EPIC algorithms between the subgroups. **(B)** Different normalized enrichment scores of immune cells between the subgroups. **(C)** Different expressions of the immune checkpoint inhibitor between the subgroups. **(D)** Differences in the ESTIMATE score between the subgroups. **(E)** Heatmap of different immune -related pathway enrichment scores between the subgroups. **(F)** Differences in response to the immune checkpoint inhibitor treatment based on the TIDE algorithm.

The heatmap of immune response based on different immune-related algorithms is depicted in **Figure 4A**. Then, ssGSEA was introduced to compare the immune cell enrichment scores between C1 and C2 (**Figure 4B**). It was found that most immune cells were all highly infiltrated in the C2 subgroup. Only neutrophil was highly infiltrated in the C1 subgroup. Then, the expression level of nine immune check inhibitor genes was compared between C1 and C2 subgroups. It was found that most of these genes (including CD274, CD276, CTLA4, CXCR4, IL6, LAG3, PDCD1, and TGFB1) were upregulated in the C2 subgroup (**Figure 4C**). Meanwhile, the R “estimate” package was utilized to investigate the immune-related scores between C1 and C2, and all the immune-related scores (including stromal score, immune score, and estimate score) were significantly higher in the C2 subgroup (**Figure 4D**). The R “GSVA” package was used to compare the immune-related signal enrichment scores between C1 and C2, and the result was consistent with the results above, indicating that all immune signals were more highly enriched in C2 (**Figure 4E**). Finally, by using the TIDE algorithm, we compared the sensitivity of the immune checkpoint inhibitors among the two subgroups and found that the response rate in the C1 subgroup was higher than that in the C2 subgroup (40.8% *vs.* 24.6%) (**Figure 4F**).

### Crosstalk Between Cancer and Immune Cells Based on Pyroptosis

**Figure 5:**
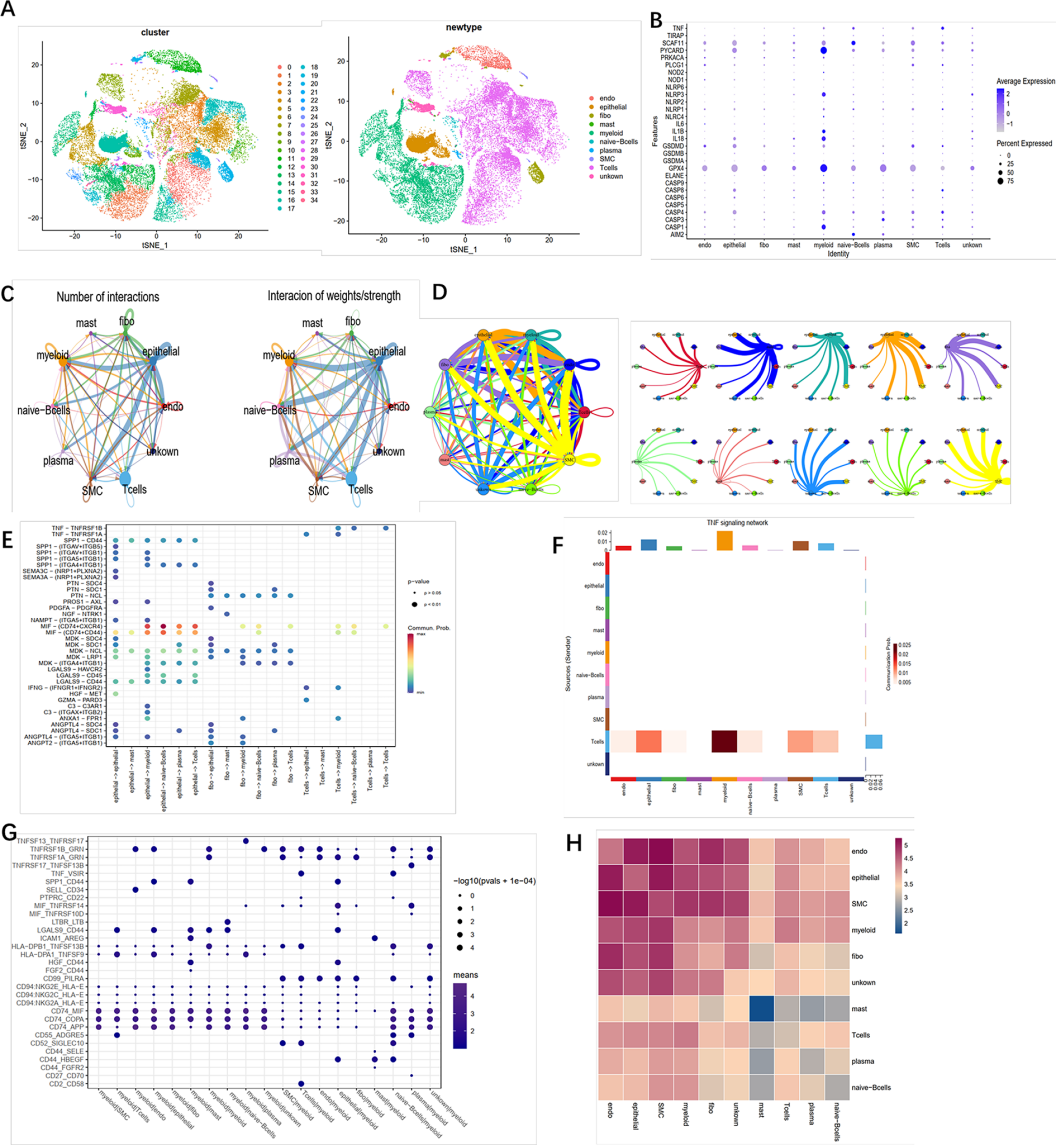
Crosstalk between cancer and immune cells. **(A)** The t-SNE plot shows that 29,799 cells were divided into 35 clusters, among which T cell accounts for the largest cluster. **(B)** Pyroptosis- related genes expressed in the 10 cell clusters. **(C)** Cell–cell interaction in cell clusters as analyzed by “CellChat.” **(D)** Cell–cell interaction in cell clusters as analyzed by “cellphonedb.” **(E)** Connection probability of main signaling pathways in cell clusters as analyzed by “CellChat.” **(F)** TNF signaling pathways between T cells and other cells. **(G)** Connection probability of main signaling pathways in cell clusters as analyzed by “cellphonedb.” **(H)** Correlation of the communication ratio between cell clusters.

To identify the role of pyroptosis-related genes in the TME of ccRCC, we collected the single cell sequence datasets from ccRCC patients who had never received any drug therapy, totally containing 29,799 cells from the dataset provide by Chirag et al. Next, nonlinear dimensionality reduction (t-distributed stochastic neighbor embedding, t-SNE) and graph-based Louvain clustering algorithm were applied to investigate the cell distribution and heterogeneity of ccRCC, which included 1,252 endothelial cells, 5,552 epithelial cells, 1,329 mast cells, 7,151 myeloid cells, 81 naive B cells, 152 plasma cells, 192 smother muscle cells, 11,909 T cells, and 2,142 unknown cells (**Figure 5A**). The expression level of pyroptosis-related genes in myeloid cells was significantly higher compared with other cells (P < 0.01) (**Figure 5B**). To investigate the impact of pyroptosis-related genes on cell–cell communication, we used “CellChat” and “cellphonedb” to analyze the communication among cells in TME and determine the complex cell–cell interaction network between cancer and immune cells (**Figures 5C, D**). All the cell–cell communications among cells were explored *via* CellChat and cellphonedb, respectively (**Figure S3A**). Next, we explored the pathways involved in pyroptosis and found that the TNF pathway (including TNFRSF1B-GRN and TNFRSF1A-GRN) could trigger pyroptosis in myeloid cells (**Figures 5E–G**). Our correlation analysis further verified the above results (**Figure 5H**). In summary, our results revealed that pyroptosis-related genes had the potential to shape the unique TME of ccRCC. Then single cell datasets from David et al. were used to compare the different expression level of pyroptosis-related gene among different stage in ccRCC, which indicated that most of pyroptosis genes were highly expressed in the tumor tissue; also the expression level of those genes was increased along with the progression of the clinical stage (**Figure S3B**).

### Comprehensive and Integrated Genomic Characterization of the Two Subgroups

**Figure 6:**
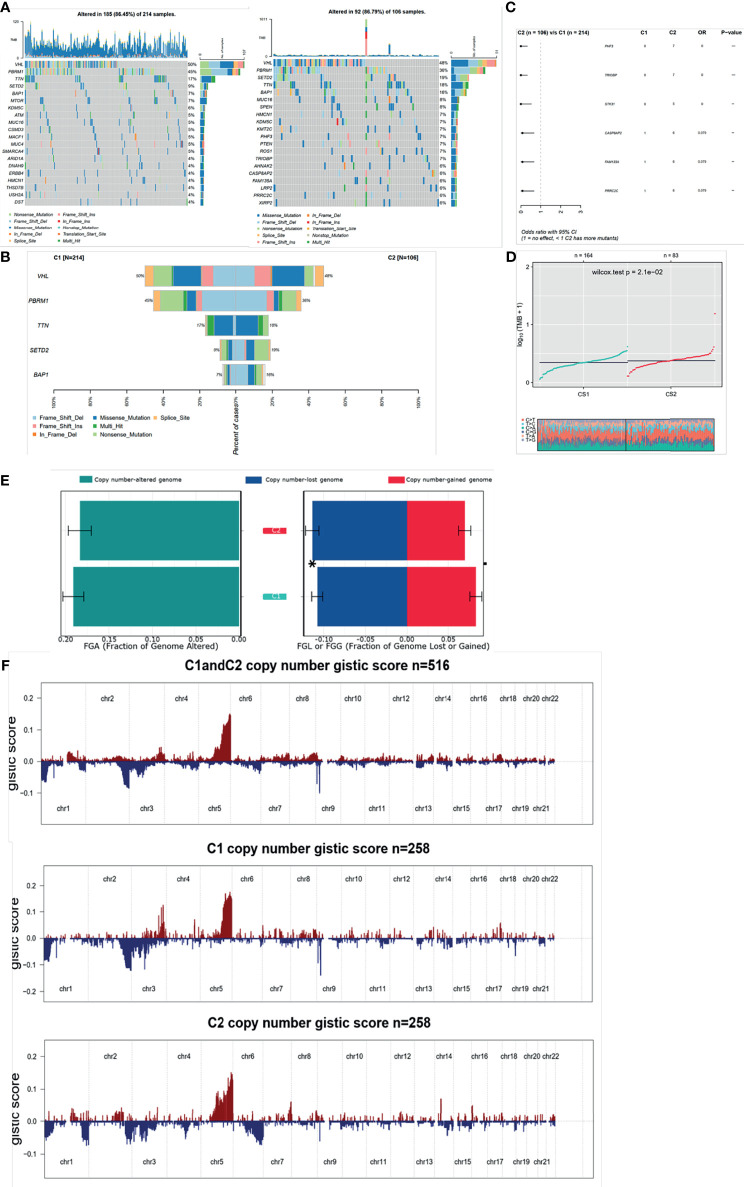
Mutation and CNV differences between subgroups. **(A, B)** Waterfall plot represents the mutation distribution of the most frequently mutated genes. **(C)** Forest plots display the top 6 most significantly differentially mutated genes between the two subgroups. **(D)** Boxplot of TMB between the two subgroups. **(E)** Barplot of fraction genome altered in the two identified subtypes. **(F)** Composite copy number profiles for ccRCC with gains in red and losses in blue and gray highlighting differences.

Since the transcriptional alterations between C1 and C2 subgroups were analyzed, then we want to investigate the disparity in the genomic layer. It was found that the mutation rate was similar between the two subgroups (185/214, 86.45% in C1 *vs.* 82/106, 77.36%). The top 20 most frequently mutated genes are depicted in **Figures 6A, B**, showing significant differences between the two subgroups. We found that most of the mutation genes were identified as protective factors in the C2 subgroup (**Figure 6C**). Also, the tumor mutation burden rate in C2 was higher than that in C1 (**Figure 6D**). Next, the CoMEt algorithm was applied to investigate the co-occurrence and exclusive mutations of the most frequently mutated genes. Compared with the pervasive co-occurrence landscape, there were unique cases in the two subgroups, which had respective unique cases that exhibited mutually exclusive mutations (**Figure S4**). Other than the mutation pattern, we also investigated differences in the copy number between the two subgroups. The GISTIC2.0 software was used to decode the amplification and deletion of CNV on chromosomes. Compared with C2, C1 had a higher copy number gained in the genome and a lower copy number in the genome (**Figure 6E**). The results showed that the C1 and C2 subgroups had frequent copy number variations (CNVs) in the region of oncogenes and tumor suppressor genes (e.g., VHL and TTN), as well as metabolic regulators (e.g., COL9A1 and COL19A1), suggesting that CNVs may play a significant role in the tumorigenesis and progression of ccRCC. The recurrent CNVs in C2 included the amplification of 5q14.3 (NR2F1-AS1), 5q33.2 (KIF4B), and 1p36.11(SYF2), as well as the deletion of 4q24 (PPA2) and 3p21.31 (LTF). The CNVs in C1 were mainly associated with tumor cell proliferation, such as the amplification of 5q31.3 (KCTD16) and 7p22.2 (SDK1), as well as the deletion of 4q24 (PPA2) (**Figure 6F**). The above results suggest that C1 and C2 subtypes had distinctive CNV events, which may cause different immune infiltrations and efficiency of target treatment in ccRCC (**Table S3**).

### Drug Sensitivity Between the Two Subgroups

**Figure 7:**
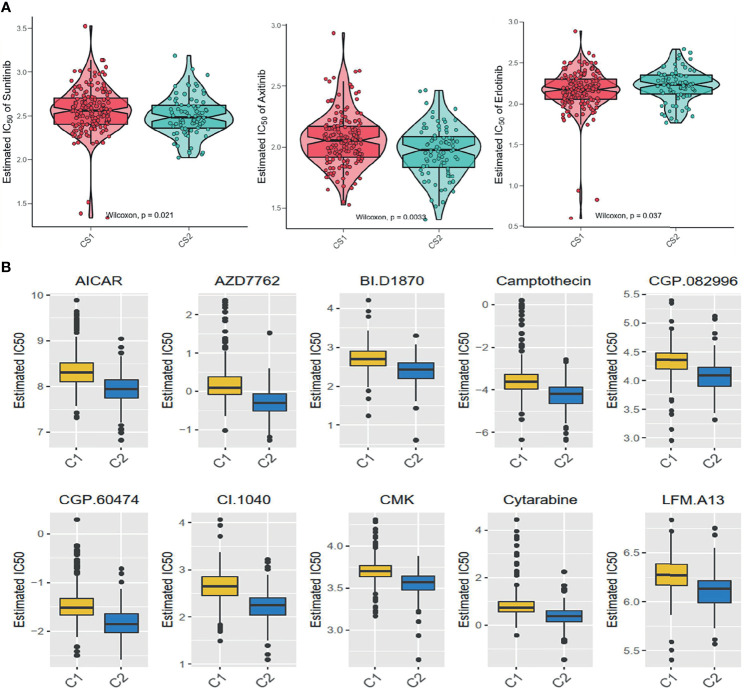
Difference of drug sensitivity. **(A)** Differences in the estimate IC50 of the molecular targeted drugs between the subgroups. **(B)** The chemotherapy response of the two prognostic subtypes to 10 chemotherapy drugs.

The GDSC database was used to forecast the chemotherapy response of the two pyroptosis subtypes to common chemotherapy drugs, which include Sunitinib, Axitinib, and Erlotinib. It was found that IC50 was significantly different between the C1 and C2 subgroups (**Figure 7A**). At the same time, several potential prodrugs with therapeutic potentialities were investigated in C1 and C2, and the results also showed different IC50 values between the two groups (**Figure 7B**). The detailed molecular structures of these drugs are shown in **Figure S5**.

### Construction and Validation of a Five-Gene Pyroptosis-Related Signature Model

**Figure 8:**
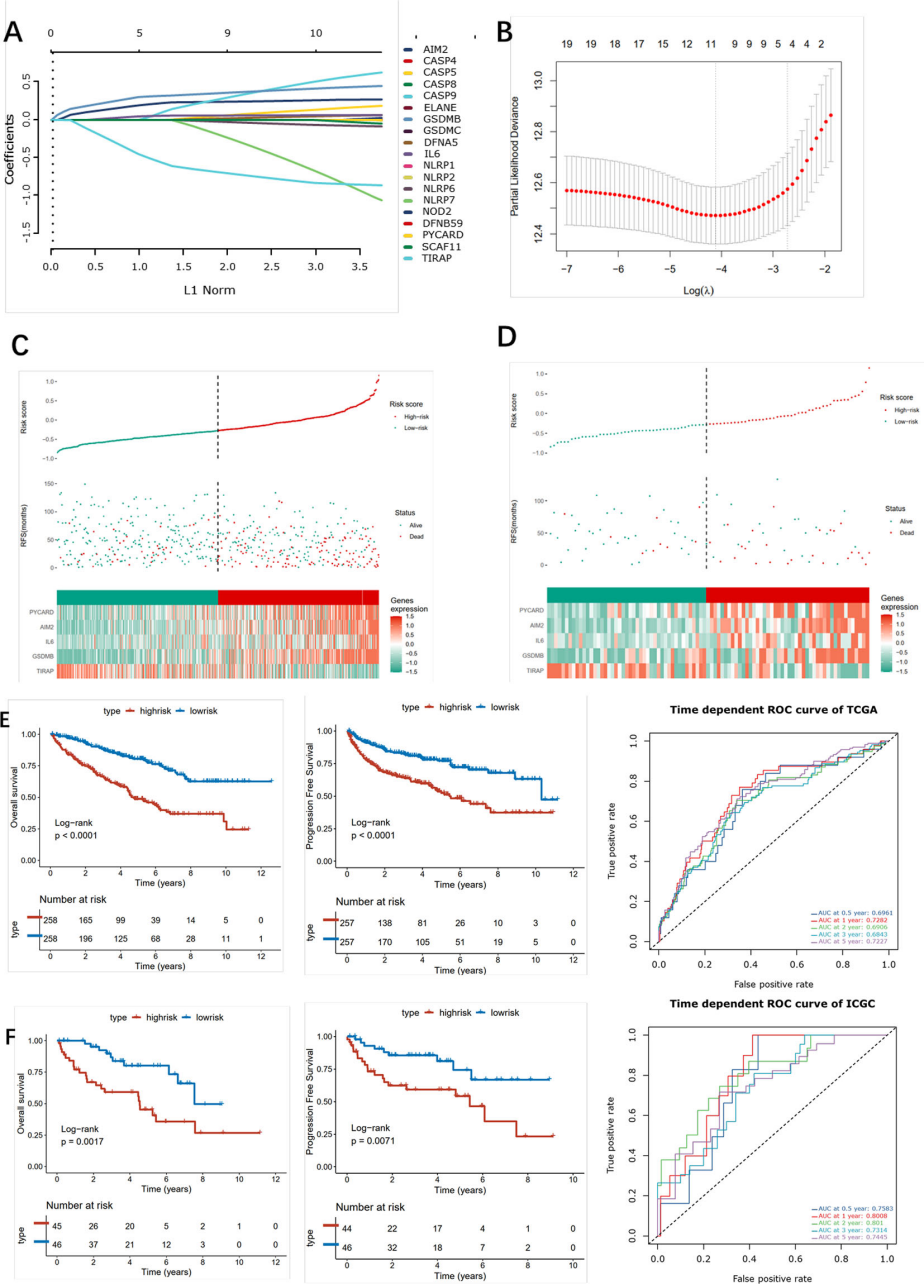
Construction of the pyroptosis -related risk scores. **(A)** LASSO coefficient plot of pyroptosis-related genes. **(B)** The optimal parameter (λ) was chosen by cross-validation. **(C, D)** Risk score analysis in ccRCC patients in the TCGA (left) and ICGC (right) cohort. **(E, F)** Kaplan–Meier analysis for OS (left) and PFI (right) of the two subtypes in the TCGA and ICGC cohort and the corresponding ROC curve.

Firstly, univariate analysis was used to select pyroptosis genes that had impact on OS. Then, the remaining 19 pyroptosis genes were further applied to Lasso-Cox regression analysis, and 10-fold cross-validation was applied to generate the optimal model. After the cross-validation, five genes, including PYCARD, AIM2, IL6, GSDMB, and TIRAP, stuck out the minimum partial likelihood deviance and reached the optimal regression efficiency (**Figures 8A, B** **).** Furthermore, a risk signature was constructed, and the risk score of each patient was calculated based on the expression levels of five pyroptosis genes: Risk score = 0.0271113*AIM2+0.04147645*GSDMB+0.01748664*IL6+0.01968024*PYCARD-0.08678271*TIRAP. To identify the pyroptosis signature responsible for OS and PFI survival prediction, TCGA–ccRCC and ICGC–ccRCC cohort samples were divided into two groups with the consideration of the median risk score (**Figures 8C, D**). It was found that patients in the high-risk group had a poorer outcome than the low-risk groups (**Figure 8E**). To determine whether the risk model had a similar prognostic value in the outer dataset, the ICGC–ccRCC cohort was used as a validation dataset, in which patients in the higher risk group got the similar outcome (**Figure 8F**). Finally, the different expression of five risk-related genes on the RT-PCR and IHC level was verified in the SMMU cohort and the Oncomine database (**Figure S6A** **).** To further evaluate the prognostic value of the cluster and riskscore subgroup in ccRCC patients, univariate and multivariate analyses were performed, which indicated that the cluster and riskscore subgroup could serve as independent risk factors, respectively, and perform better than AJCC and Grade on evaluating the long-term survival rate **(** **Figure S6B** **).** Furthermore, the cluster and riskscore subgroups showed a high internal consistency, in which C1 was mainly included in the riskscore low subgroup, while C2 was included in the riskscore high subgroup (**Figure S6B** **).**

## Discussion

Pyroptosis, which is recognized as a special type of programmed cell death, has been proven to participate in oncogenesis, progression, immune infiltration, and antitumor response (22). The role of pyroptosis in urologic carcinoma has attracted increasing attention in recent years. In this study, we firstly analyzed the role of pyroptosis in ccRCC and found that pyroptosis-related genes were differentially expressed between ccRCC and normal renal tissues. Then, we identified two heterogeneous pyroptosis-related subgroups (C1 and C2) in ccRCC patients by unsupervised cluster algorithms. It was found that the C1 subgroup possessed a high level of pyroptosis-related genes and high abundance of immune cells, which was defined as the pyroptosis high and immunity high subtype. C2 expressed low pyroptosis genes and lacked infiltrating immune cells, which was defined as the pyroptosis low and immunity low subtype. In addition, we constructed a pyroptosis-based risk model based on the pyroptosis signature and evaluated its accuracy and stability in validation datasets, hoping that the results obtained would help better understand the pyroptosis role in ccRCC and promote precise therapy of ccRCC.

It was found in this study that the two subgroups had distinctive clinical characteristics. Patients in the C1 subgroup had better OS and PFI relative to C2, and patients in the C2 subgroup were associated with worse clinical characteristics in terms of the stage and grade. We further analyzed the drug sensitivity between the two subgroups and found that the IC50 in C1 was higher than that in C2 under the treatment of bicalutamide and afatinib, while patients in the C2 subgroup were more sensitive to imatinib, lisitinib, gefitinib, and sunitinib. In addition, compared with C2, patients in the C1 subgroup obtained more benefits from immune therapy as compared with those in the C2 subgroup.

Next, we investigated genome alterations in C1 and C2 subtypes and found that that SPEN was a particular SMG of C2 and associated with the formation of centrosomes and cilia, and the loss of centrosomes was required for the formation of an apoptotic microtubule network. Based on the above evidence, we speculated that SPEN loss in ccRCC may induce pyroptosis formation. SETD2, which is a histone H3K36 methyltransferase, participates in gene transcription and DNA repair by regulating chromatin biology (23). Intriguingly, recent research found that SETD2 could methylate tubulin and STAT1 and thus influence cytoskeleton remodeling and interferon response, respectivly (24). It was found in our study that the mutation frequency in the C2 subgroup was higher than that in C1. Until now, there has been no research about SETD2 mutation with pyroptosis, and we speculate that SETD2 could enhance pyroptosis in ccRCC.

Additionally, we explored the relationship between ccRCC TME and pyroptosis. Compared with C1, C2 presented more accumulation of immune cell infiltration in TME and higher immune activity. In addition, the expression of the immune checkpoint inhibitor genes was also higher than that in C2. All these findings suggest that the response for immune checkpoint inhibitor therapy in C1 was significantly higher than that in C2. Given the high pyroptosis status in C2, we speculate that even pyroptosis could recruit immune cell infiltration in TME but build a disturbed and suppressive state in TME, suggesting that targeting pyroptosis may convert the disturbed and suppressive state and thus enhance the immunotherapy effect. ccRCC was a distinctive tumor infiltrated with high densities of CD8 T cells while having poor prognosis, indicating that there might be more complicated mechanism in ccRCC. Zhang and Wang et al. found that tumor cells could recruit tumor-suppressed immune cells *via* uncontrollable pyroptosis, thus weakening the immune checkpoint inhibitor efficency (25, 26). Based on the evidence above, we speculated that pyroptosis could enhance the immune suppressive immune microenvironment and thus assist the progression of ccRCC.

Tumorigenesis is a mutagenic process involving the participation of the TME component. Recent studies indicated the necessity of cell-to-cell communications in the progression of solid tumors (27). In the above study, we had identified two pyroptosis status subgroups in ccRCC, while the data came from bulk sequence, and it is therefore urgent to analyze the role of pyroptosis-related genes in a single cell level. Interestingly, we found that the pyroptosis-related genes were highly expressed in myeloid cells but not in epithelial cells (mainly including cancer cells). With the application of cell communication packages, T and epithelial cells were found to trigger the pyroptosis effect in myeloid cells *via* the TNF signaling pathways. Combined with previous immune microenvironment analysis, we concluded that TNF signaling pathways may play an important role in ccRCC, in which epithelial and T cells function as the signal sender while myeloid cells function as the signal receptor, thus further mediating the immune impressive environment *via* activating pyroptosis in myeloid cells. Tumor infiltrating T cells have perpetually been a hot spot within the field of tumor immunity. The degree of T cell infiltration in most tumors indicates a good prognosis; however, there are diametrically opposite effects in ccRCC (5). In addition, ccRCC patients have limited benefits from ICB therapy, even with no effect, which suggests that there might be distinctive regulation in the tumor microenvironment of ccRCC (27). Meanwhile, with the in-depth study of tumor infiltrating B cells and the concept of TLS, a new direction is provided for tumor immunotherapy (28). The density and composition of TLS are related to various biological characteristics of tumors. Giraldo et al. found that the degree of TLS enrichment in ccRCC was negatively correlated with the degree of infiltration of exhaustive T cells (29); Catherine et al. found that the degree of TLS infiltration in most tumors was positively correlated with a good prognosis, and TLS mainly contains myeloid cells and plasma cells (30). Several studies have shown that TLS and killer T cells play a synergistic effect and thus produce strong antitumor immune effects; Harris et al. found that plasma cells and T cells in TLS cooperate with each other in breast cancer, and CD8+ T cells could be a protective factor only in the presence of plasma cells (31). In addition, the TNF-TNFRSF axis plays an important role in the maintenance of TLSs. David et al. found that TNFRSF17 could promote the infiltration of plasma cells in TLSs in ovarian cancer, thereby promoting antitumor immunity (32). Our study found that the TNF-TNFRSF-related network influences the ccRCC microenvironment, and T cells act as signal transmitters to activate the TNF signal axis of myeloid cells. However, with the progression of ccRCC, the communication intensity gradually decreases, which indicates that TNF signal communication in the immune microenvironment of ccRCC could effectively inhibit the progression of kidney cancer. Based on the evidence we found in this study, it is likely that it might reverse the immune impressive state in ccRCC by stimulating the TNF signal inhibitor while inhibiting the pyroptosis in myeloid cells. However, this hypothesis needs to be tested, and these experiments are underway in our laboratory.

Recently, several studies found that the pyroptosis signal might play a dual role in tumor development and drug response (33). On one hand, long time exposure to inflammation could facilitate tumorigenesis; on the other hand, pyroptosis could inhibit the development of cancers. As the activator in the pyroptosis signal, the TNF family participates in various diseases *via* influencing pyroptosis and/or apoptosis. Wang et al. found that chemotherapy and TNF could induce pyroptosis *via* caspase3 in GSDME highly expressed cell line (34). At present, the effect of pyroptosis-related genes in ccRCC is still unclear. Darren et al. found that the DPP8/DPP9 inhibitor could induce pyroptosis in acute myeloid leukemia *via* CARD8 (35). Our study found that the expression of most pyroptosis-related genes positively correlated with the progression of ccRCC, which was significantly expressed in the myeloid cell subpopulations of ccRCC. This result suggests that the activation of the pyroptosis signal in myeloid cells might promote the development of ccRCC. In summary, we found that although TNF plays an important role in promoting TLSs and tumor infiltration of B cells in tumors, this axis can simultaneously activate the pyroptosis pathway of myeloid cells, thereby inhibiting the antitumor response of myeloid cells. In addition, we speculate that it is promising to facilitate the therapeutic effect of ccRCC by maintaining the formation of TLSs while inhibiting pyroptosis in myeloid cells.

Furthermore, the pyroptosis status may also have an impact on sensitivity to chemotherapy in ccRCC. According to the estimate IC50, patients in C1 may be more sensitive to Bicalutamide and Afatinib, while C2 may be sensitive to Imatinib, Lisitinib, Gefitinib, and Sunitinib. Based on the pyroptosis status, medical care workers can choose a suitable treatment scheme for patients more accurately. Since the poorer prognosis and lower sensitivity to drug therapy in C2, we used the GDSC database to identify small-molecule drugs for C2 patients, including some anticancer drugs such as AICAR, Camptothecin, and Cytarabine. AICAR (5-aminotimidazole-4-carboxamide riboside or acadesine) is an agonist targeting at AMP mediate kinase, which can inhibit several cancer cell survivals *via* inducing cytotoxic effect. Liang et al. found that the combined use of AICAR and Rapamycin could effectively reduce cell proliferation, increase cell apoptosis, and markedly decrease the level of HIF-2α, p-Akt, and vascular endothelial growth factor expression in the renal tumor (36). Camptothecin is a natural anticancer drug in traditional Chinese medicine. Xiao et al. found that the Camptothecin analogue G2 could induce apoptosis in liver cancer and colon cancer cell lines by inducing ROC accumulation and reducing MMP (37). Galley et al. reported two types of Camptothecin analogues (CPT-11 and 9-AC), which showed a remarkable survival extension in an orthotopic model of late-stage renal cancer tissue (38). Cytarabine is an effective drug in the treatment of certain hematologic malignancies. Song et al. found that a new generation of cytarabine (Ara-C) analogs could induce the apoptosis effect in prostate cancer *via* targeting MK2 and inducing the synergic antitumor effect in p53-deficient prostate cancer cells combining with cabozantinib (39). Hence, these candidate molecular drugs might also possess potential efficacy for ccRCC.

In this study, we also constructed a pyroptosis-related gene risk model that could precisely predict the OS and PFI of ccRCC patients in training and validation cohorts. Among the risk model, IL6, an essential cytokine, functions as a moderator participating in inflammation and maturation of B cells. IL6 is involved in the STATS-mediated signal transduction pathway by mediating tumor immune suppression, tumor cell survival, premetastatic niche formation, and chemotherapy resistance. Yang et al. found that CS-Iva-Be, which is a special IL6R antagonist, inhibited the IL6/STAT3 pathway and sensitized breast cancer cells to TRAIL-induced cell apoptosis (40). We found that high IL6 expression was a risk predictor in survival outcome in ccRCC. GSDMB is a member of the gasdermin (GSDM) family, which can adopt completely different mechanisms of building-block domain interactions to modulate their lipid-binding and pore-forming activities. The GSDM family participates in cell proliferation and differentiation, particularly *via* regulating pyroptosis. Several studies on solid cancers have verified that GSDMB is extremely expressed in cancer tissues including uterine, cervical, breast, and stomachic cancers (41). In this study, we found that GSDMB was highly expressed in ccRCC, and this high expression was positively correlated with ccRCC progression. TIRAP is a member belonging to the TLR/IL-1R family, and an aberrant expression of TIRAP could induce tumorigenesis including gastric cancer, colorectal cancer, and lymphocytic leukemia (42). It was also found that phycocyanin, a food-derived inhibitor, could inhibit TIRAP in NSCLS cells and exert an antiproliferation effect through downregulating TIRAP/NF-kB activity in lung cancer (42). We found that phycocyanin could also serve as a protective factor in ccRCC, and its high expression correlated with the poor outcome of ccRCC patients. AIM2 comes from interferon-inducible PYRIN and HIN domain-containing family. Recent studies revealed that AIM2 functions as a DNA sensor and participates in innate immunity *via* binding to foreign double-stranded DNA in host macrophages (43). AIM2 could trigger the assembly of inflammasomes to induce a caspase1-mediated inflammatory response, causing cell apoptosis. Chen et al. found that exogenous AIM2 expression could attenuate cancer cell proliferation *via* inhibiting NF-κB activity, thus suppressing mammary tumor growth in breast cancer (44). Our study found that AIM2 was highly expressed in renal tumor tissues compared with normal tissue, and its high expression indicates poor prognosis of ccRCC patients. PYCARD is a pro-apoptotic protein, participating in the moderation of programmed cell death. Miao et al. found that an lncRNA antisense to PYCARD exhibited a dual nuclear and cytoplasmic distribution and promoted the proliferation of cancer cell lines (45). Several studies found that PYCARD was a tumor-inhibiting factor in that it was silenced in many tumor types, and the level of methylation in its promoter was negatively correlated with tumor progression (46). We found that PYCARD was highly expressed in tumor cells, and its expression was positively correlated with renal tumor progression.

Meanwhile, there are still two major limitations that require further exploration. Firstly, we only analyzed the prognostic role of the pyroptosis-related genes in ccRCC, but how those pyroptosis genes interact with each other and which pathway is involved in pyroptosis in ccRCC need further study. Secondly, although we performed an independent internal validation, it is difficult to cover all variations in patients from different race and region; thirdly, even if our study verified the different expression level of risk model-related genes on the RT-PCR and IHC level, detailed biological experiment would make our study more comprehensive. Thus, findings in our study are waiting for further well-designed experiment. Yet, despite the above limitations, there is no denying that our study is the first comprehensive study of pyroptosis-related genes in ccRCC and to identify two distinctive ccRCC subgroups on multi-omics; also the cluster and riskscore could function as an independent indicator for the evaluation of prognosis in ccRCC patients.

In conclusion, this was the first study to comprehensively investigate the role of pyroptosis in ccRCC, to identify two pyroptosis status subtypes of ccRCC, and to establish a robust pyroptosis prognostic model of ccRCC. Different pyroptosis status ccRCC displayed distinct heterogeneity in multiple levels including functional status, tumor microenvironment, alternation in genomics, response to chemotherapy and immunotherapy, and clinical outcomes. The risk model based on pyroptosis reached a satisfactory prediction ability in the OS and PFI of ccRCC. All the findings in our study could facilitate a better understanding in pyroptosis and the precise management of ccRCC patients.

## Data Availability Statement

The data presented in the study are deposited in the TCGA, European Genome-phenome Archive (EGA) and GEO repository, accession number are TCGA-KIRC, EGAS00001000509 and PRJNA705464, respectively.

## Ethics Statement

The studies involving human participants were reviewed and approved by the Ethics Committee of Changzheng Hospital. The patients/participants provided their written informed consent to participate in this study.

## Author Contributions

AJ, JM and YB have contributed equally to this work. JL, BL, and LW conceptualized and designed this study. AW, WG, XG, JW, YB and ZW wrote the first draft of the manuscript. All authors contributed to the article and approved the submitted version.

## Funding

This study was funded by the National Natural Science Foundation of China (82072812, 81730073, and 81872074), Shanghai Sailing Program (19YF1448300), and Clinical science and technology innovation project of Shanghai Shenkang Hospital Development Center (SHDC12018108).

## Conflict of Interest

The authors declare that the research was conducted in the absence of any commercial or financial relationships that could be construed as a potential conflict of interest.

## Publisher’s Note

All claims expressed in this article are solely those of the authors and do not necessarily represent those of their affiliated organizations, or those of the publisher, the editors and the reviewers. Any product that may be evaluated in this article, or claim that may be made by its manufacturer, is not guaranteed or endorsed by the publisher.

## Supplementary Material

Supplementary Table 1 Information of 33 pyroptosis-related genes

Supplementary Table 2 Primer sequences of 5 hub genes.

Supplementary Table 3 CNV events among C1 and C2.

Supplementary Figure 1 Workflow of this study.

Supplementary Figure 2
**(A)** Enrichment analysis of DEGs; **(B)** Transcription factor analysis of dysregulated genes; the purple nodes represent down-regulated genes(left) and un-regulated genes(right), and green nodes represent transcription factors; **(C)** Consensus cluster matrix of ccRCC tumor samples when k =2, and the cumulative distribution function (CDF) in ICGC cohort(left); The expression heatmap of the 33 pyroptosis-related genes in the two subtypes(middle); Kaplan-Meier survival curve of predicted subtype of ccRCC in ICGC database (right).

Supplementary Figure 3
**(A)** Heatmap showing the selected ligand-receptor interactions between cancer cells and other cells *via* CellChat; **(B)** Bubble plot showing the selected ligand-receptor interactions between cancer cells and other cells *via* cellphonedb.

Supplementary Figure 4 Heatmap illustrating the co-occurring and exclusive mutations of the top 25 frequently mutated genes.

Supplementary Figure 5 The structure tomographs of ten candidate small-molecule drugs.

Supplementary Figure 6
**(A)** Different expression level of five risk related genes in SMMU cohort and Oncomine database. **(B)** Univariate and multivariate analysis of overall survival of ccRCC patients; Time dependent ROC curve on AJCC, Grade, cluster and riskscore subgroups in ccRCC patients; Consistency heatmap of cluster and riskscore subgroups; test of equal scale model of multi variates using in Univariate and multivariate analysis.
