# Supplementary material for: Establishment of a prognosis Prediction Model Based on Pyroptosis-Related Signatures Associated With the Immune Microenvironment and Molecular Heterogeneity in Clear Cell Renal Cell Carcinoma
Source: Front Oncol. 2021 Nov 5;11:755212. doi: 10.3389/fonc.2021.755212 (PMC8603037; doi:10.3389/fonc.2021.755212)
Supplement: Supplementary Table 1 — Information of 33 pyroptosis-related genes [file DataSheet_1.zip › Supplementary Table 2.docx]

PYCARD

F: TGGATGCTCTGTACGGGAAG

R: CCAGGCTGGTGTGAAACTGAA

AIM2

F: TGGCAAAACGTCTTCAGGAGG

R: AGCTTGACTTAGTGGCTTTGG

IL6

F: ACTCACCTCTTCAGAACGAATTG

R: CCATCTTTGGAAGGTTCAGGTTG

GSDMB

F: TGATTGCCGTTAGAAGCCTTG

R: TCCCGTTGAGTCTACATTATCCA

TIRAP

F: ATGGCATCATCGACCTCCCT

R: GTCACTCGCATGTGTGGGT
