# Supplementary figures and images for: Establishment of a prognosis Prediction Model Based on Pyroptosis-Related Signatures Associated With the Immune Microenvironment and Molecular Heterogeneity in Clear Cell Renal Cell Carcinoma
Source: Front Oncol. 2021 Nov 5;11:755212. doi: 10.3389/fonc.2021.755212 (PMC8603037; doi:10.3389/fonc.2021.755212)

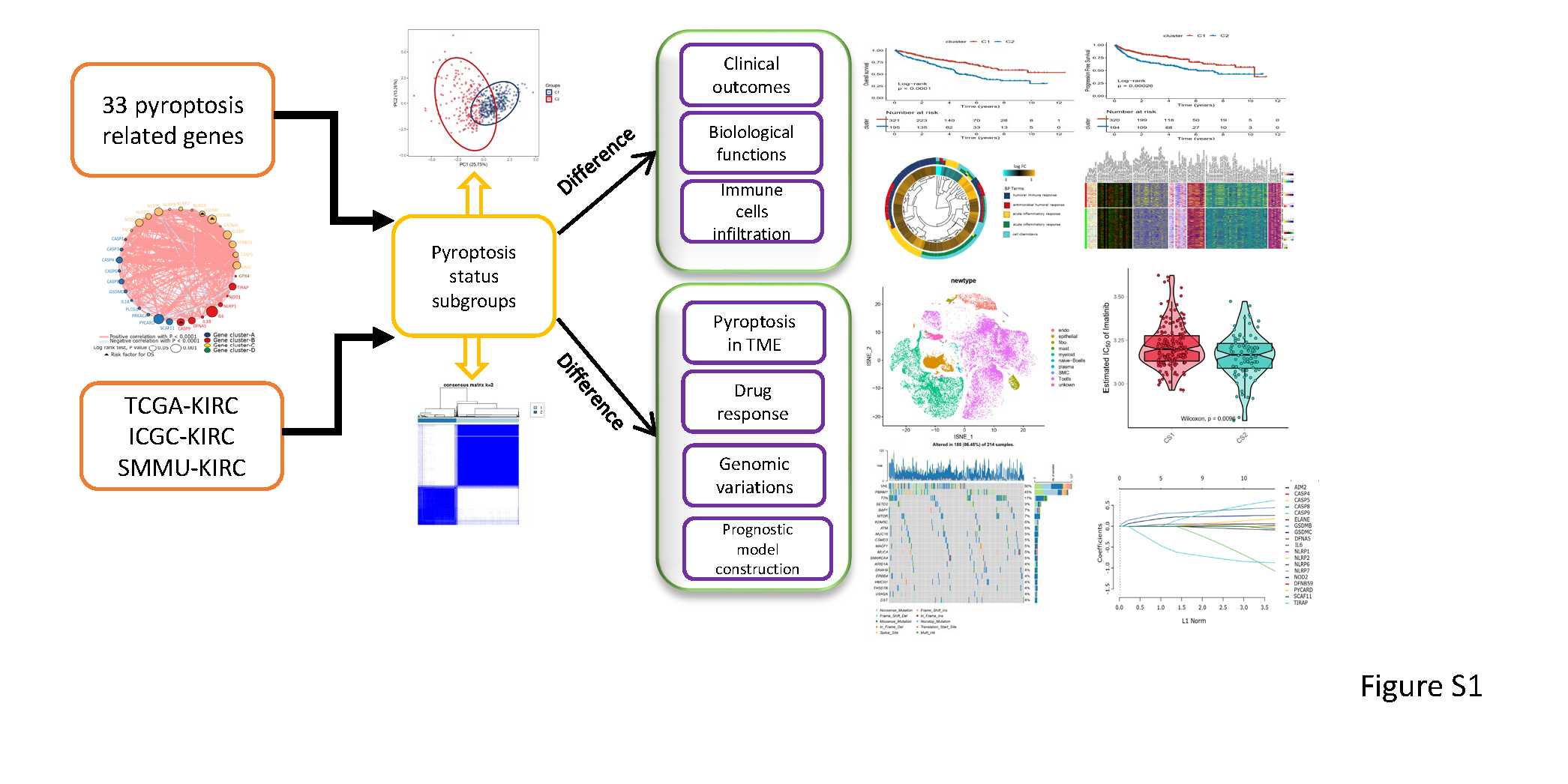

Supplement: Supplementary Table 1 — Information of 33 pyroptosis-related genes [file DataSheet_1.zip › Supplementary Figure 1.tif]

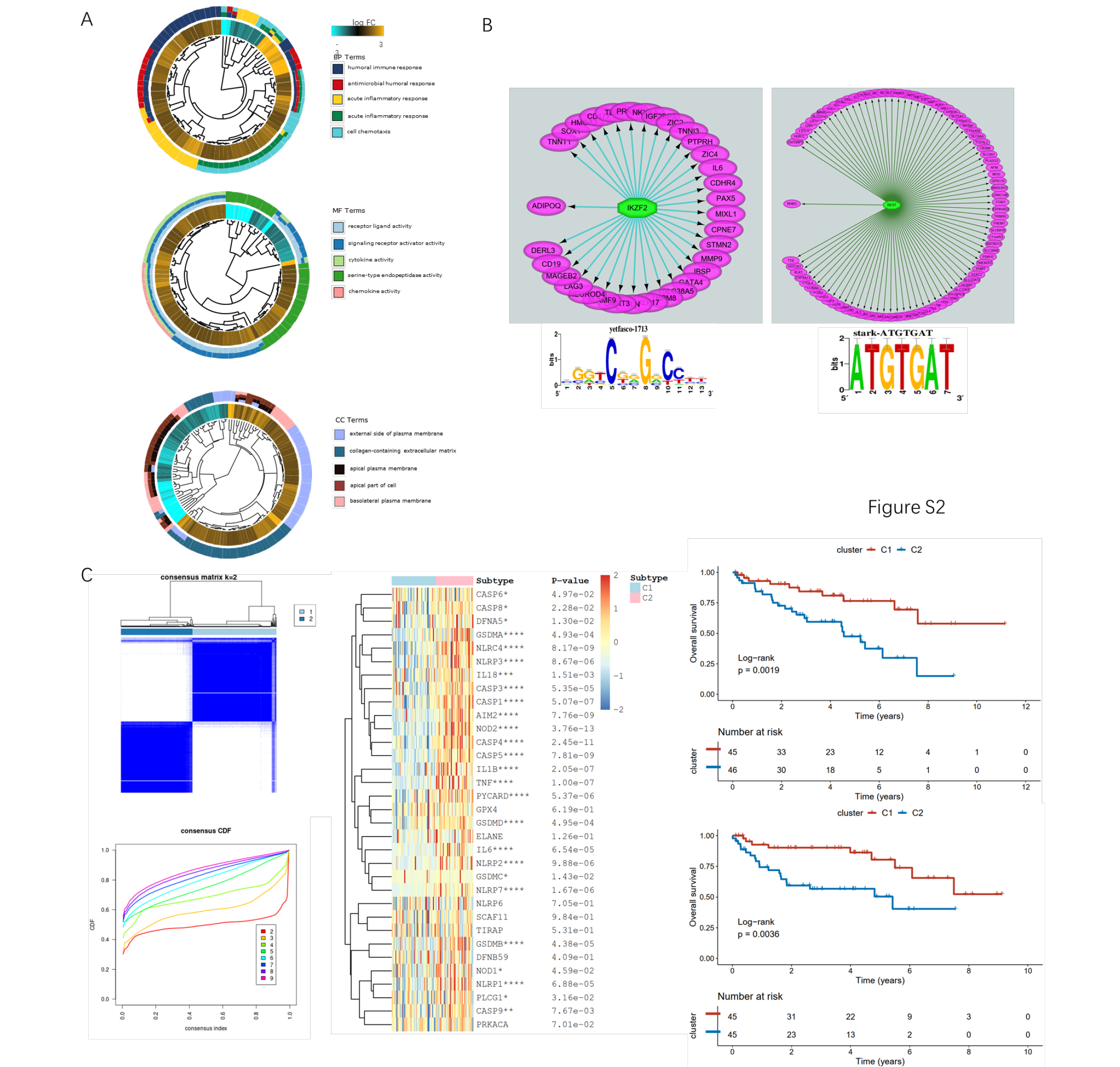

Supplement: Supplementary Table 1 — Information of 33 pyroptosis-related genes [file DataSheet_1.zip › Supplementary Figure 2.tiff]

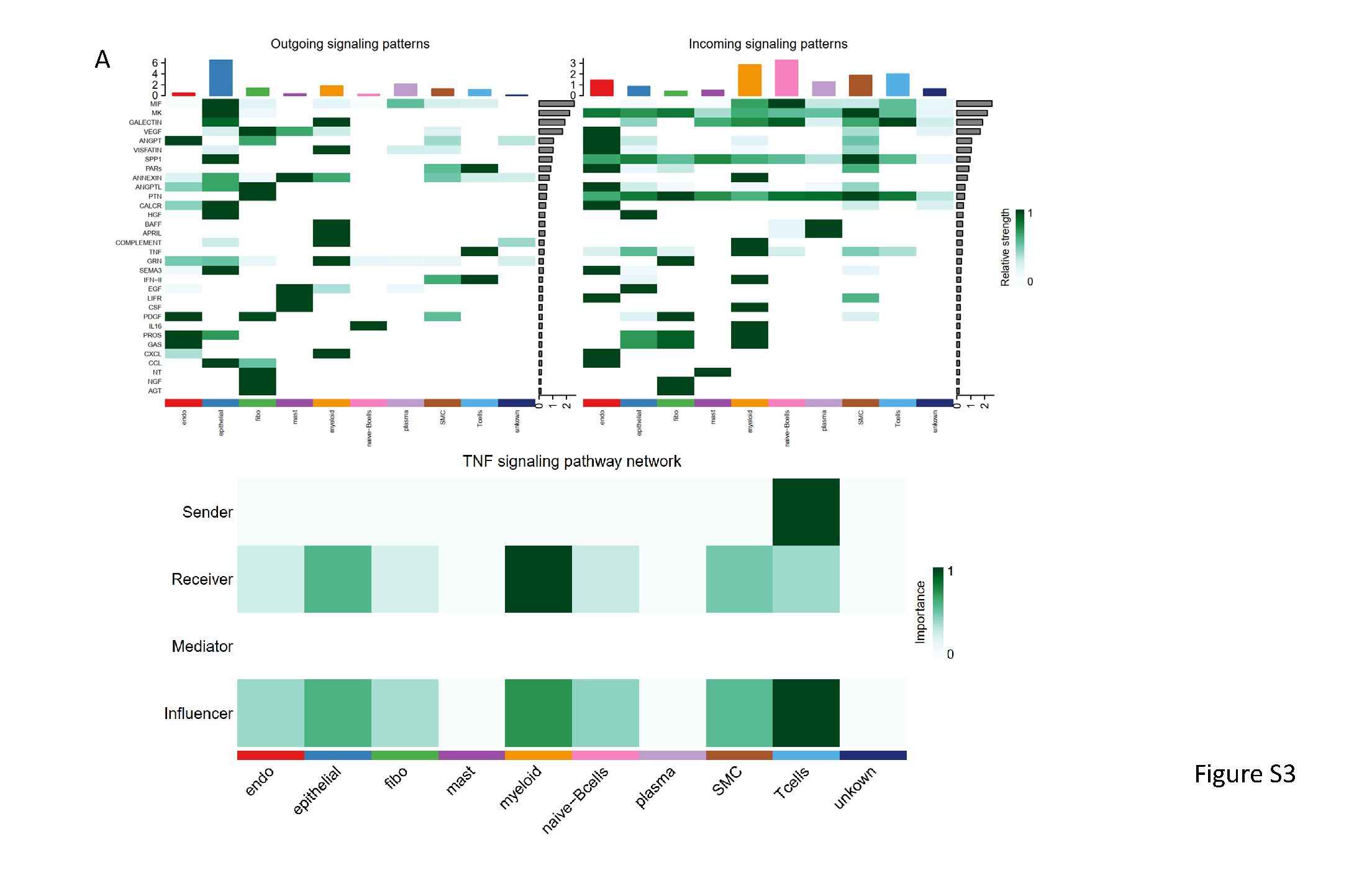

Supplement: Supplementary Table 1 — Information of 33 pyroptosis-related genes [file DataSheet_1.zip › Supplementary Figure 3(A).tif]

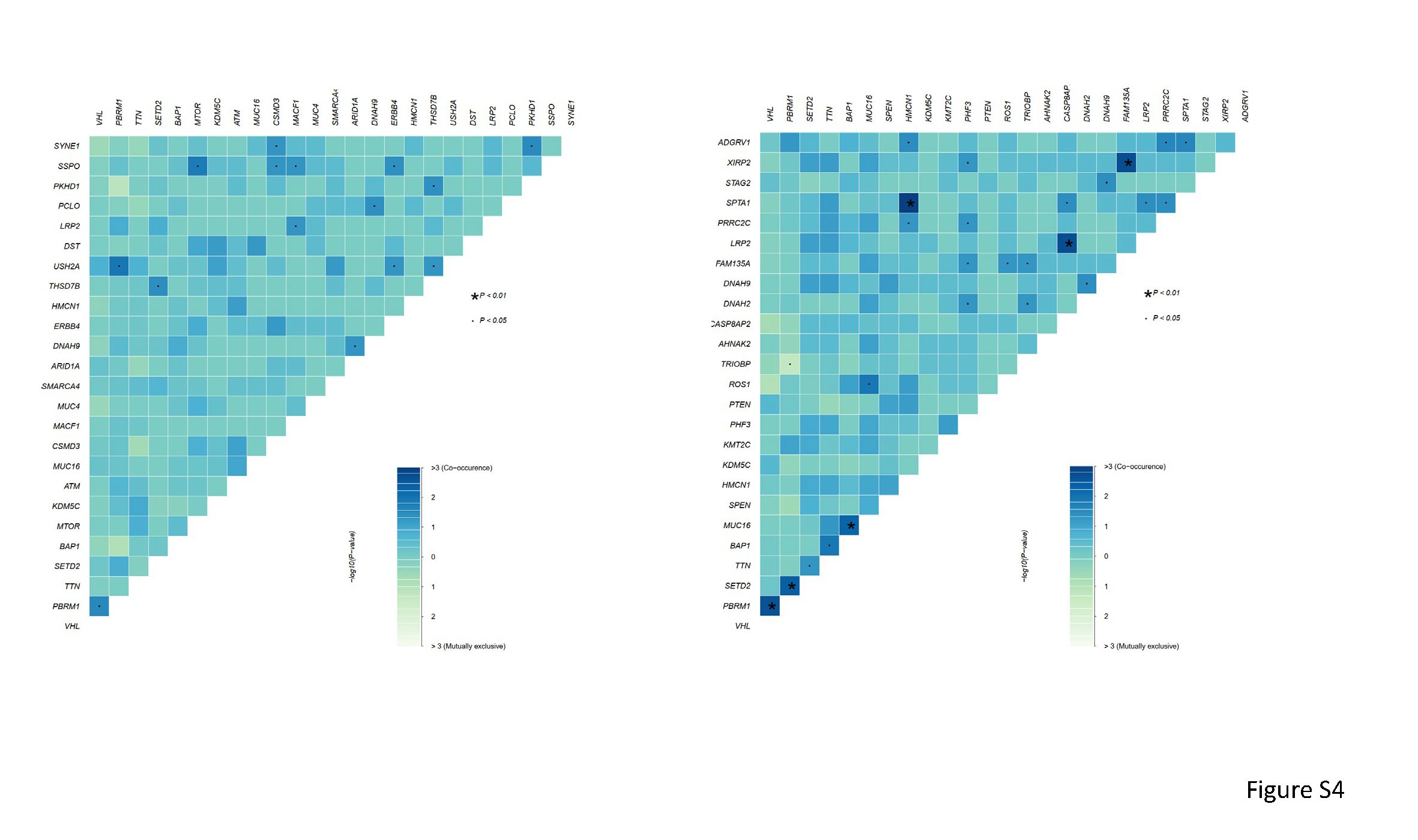

Supplement: Supplementary Table 1 — Information of 33 pyroptosis-related genes [file DataSheet_1.zip › Supplementary Figure 4.tif]

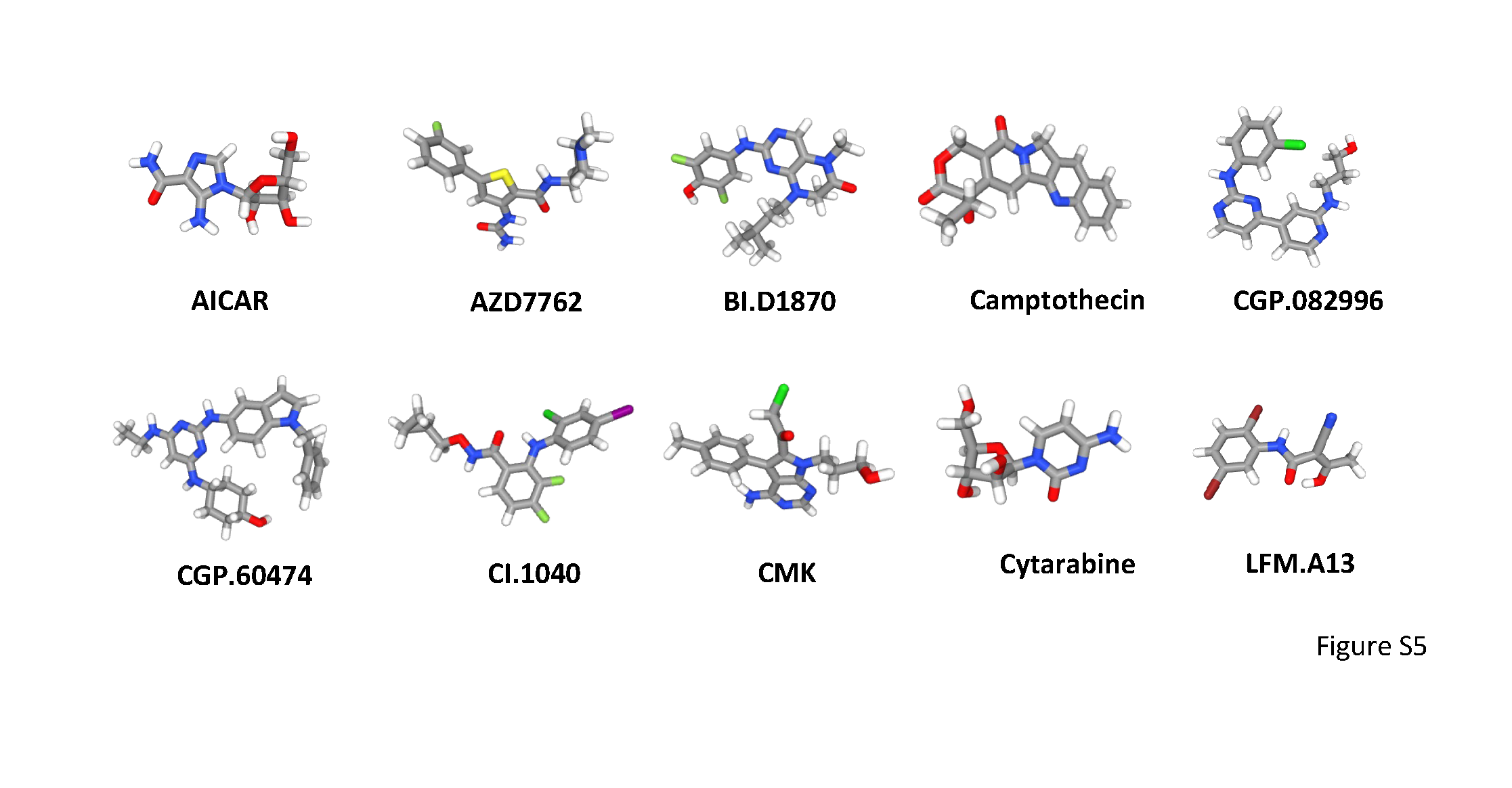

Supplement: Supplementary Table 1 — Information of 33 pyroptosis-related genes [file DataSheet_1.zip › Supplementary Figure 5.tif]

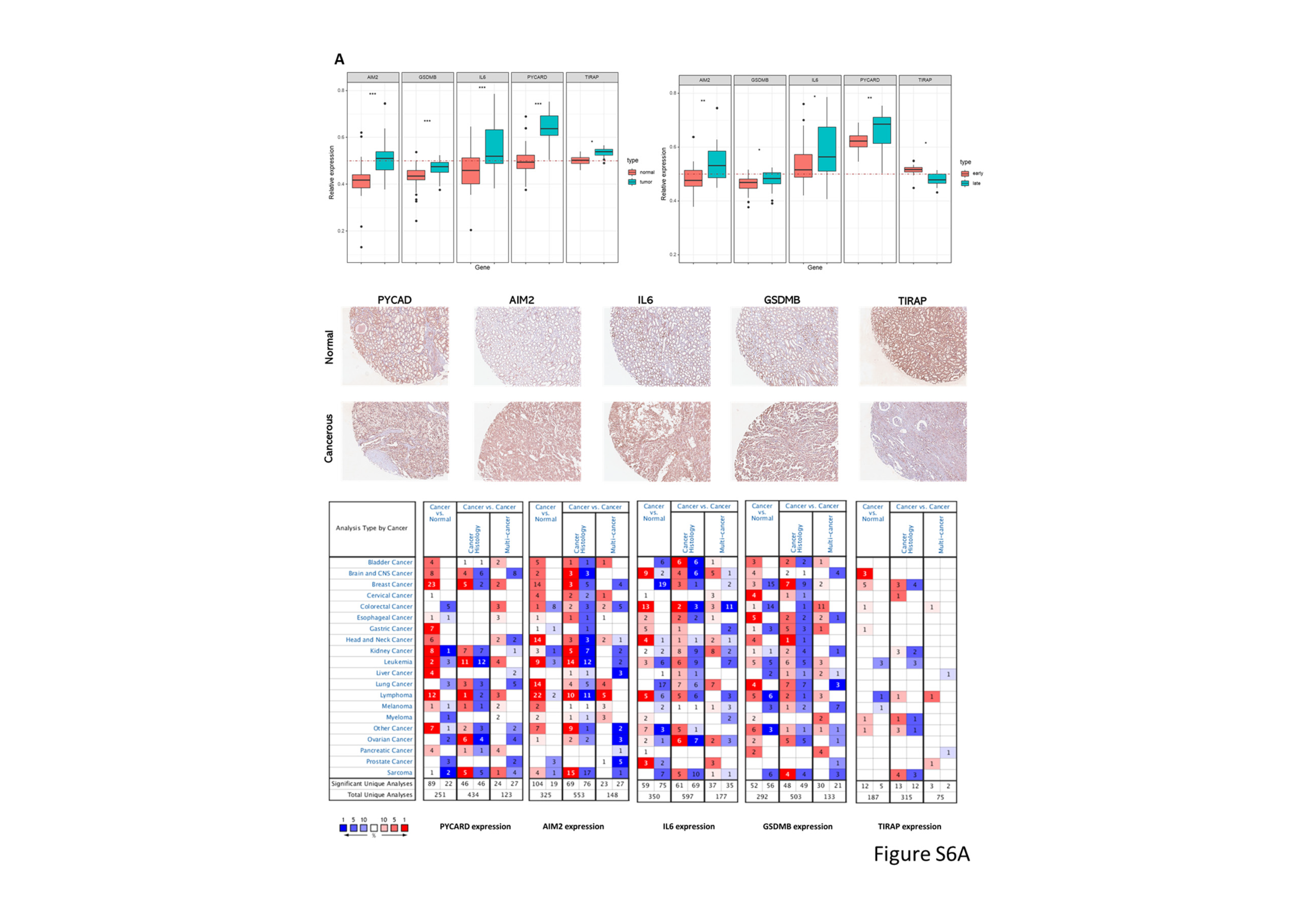

Supplement: Supplementary Table 1 — Information of 33 pyroptosis-related genes [file DataSheet_1.zip › Supplementary Figure 6 (A).tiff]

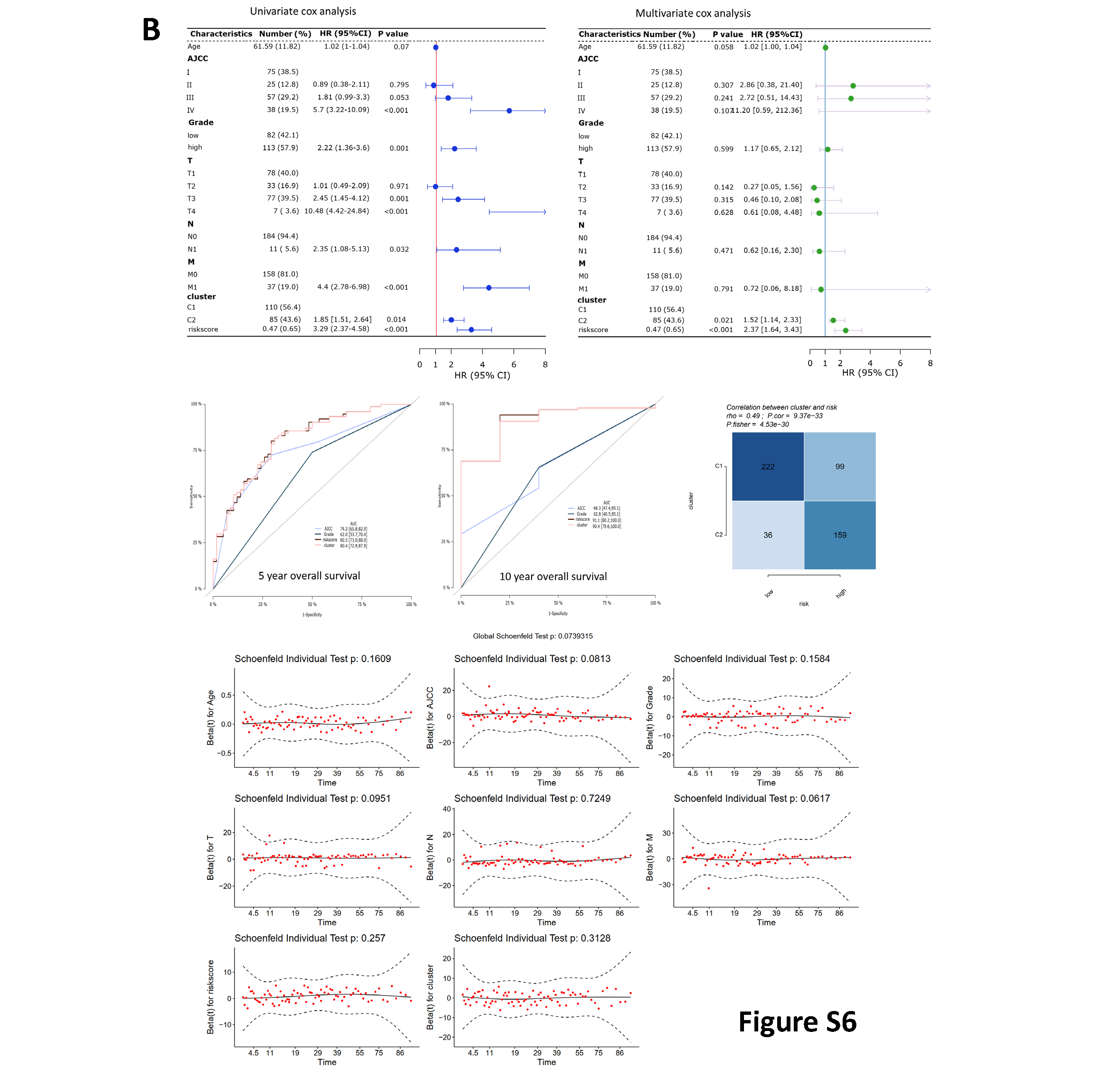

Supplement: Supplementary Table 1 — Information of 33 pyroptosis-related genes [file DataSheet_1.zip › Supplementary Figure 6 (B).tif]
