# Supplementary material for: Establishment of a prognosis Prediction Model Based on Pyroptosis-Related Signatures Associated With the Immune Microenvironment and Molecular Heterogeneity in Clear Cell Renal Cell Carcinoma
Source: Front Oncol. 2021 Nov 5;11:755212. doi: 10.3389/fonc.2021.755212 (PMC8603037; doi:10.3389/fonc.2021.755212)

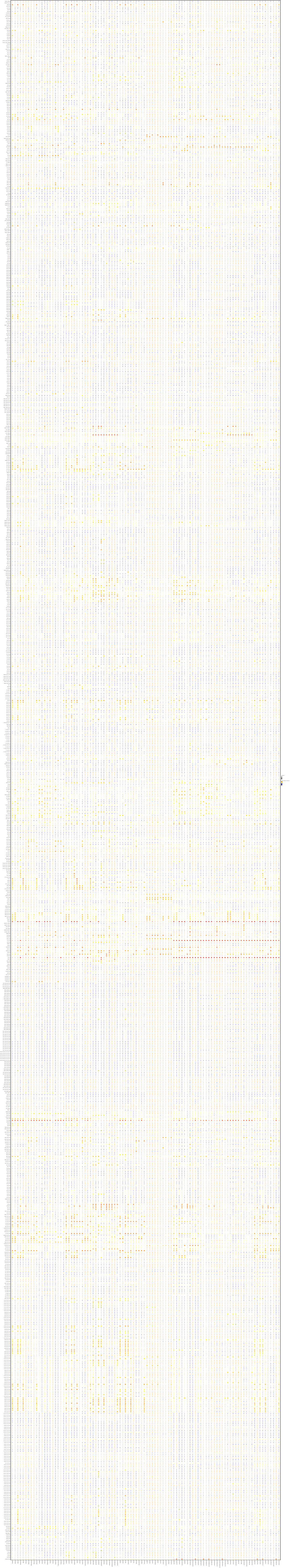

Supplement: Supplementary file 2 [file DataSheet_2.pdf]
